# 

**Published:** 1896-01-18

**Authors:** 


					THE HOSPITAL. t ABSOLUTELY PURE, therefora BEST. January 18th, iss>6.
CADBURY'S ^ ww ^ CADBURY'S
COCOA IHf COCOA
The standard of highest purity at present NO ALKALIES USED,
attainable, ?Laneet.
Q. O
CV , , , ,  -   --
flSC7)ir Western I HFIRNARy^^ Z^SiZg-?^ :  ..-USI - -iWftTiUfAuaNUflW/CH HOSPITAL
No. 486; Vol. XIX.
Saturday,
Jan. 18th, 1896.
WITH EXTRA NUR8ING SUPPLEMENT. ?ce twopence.
[Registered at the General Post Offioe as a Newspaper for Monthly Parts, 6d.j "i^t frw!*8d.
transmission in the United Kingdom and Abroad.] Ditto per Annum, 8s.6d., post free!

				

